# Detection of Human Blood

**Published:** 1889-10-12

**Authors:** 


### DETECTION OF HUMAN BLOOD.

A "Report on the Medico-Legal Detection of Human Blood" has appeared by Dr. Monckton Copeman, demonstrator of physiology and of morbid histology at St. Thomas's Hospital.* Up to the present time the only method of distinguishing human blood was the detection and measurement of the red corpuscles. But it would be unsafe to testify before a jury that even fresh blood was human, if only this plan were followed. Much more would the results be open to criticism in the case of corpuscles derived from a dried stain, since it would be impossible to say that their size had not been altered by the solvent employed. What has been stated applies to mammalian blood, for the corpuscles of the blood of birds, fishes, reptiles, or amphibians, are oval and nucleated, and may be obtained without alteration of their characteristic shape. In 1887 the author published some papers on the pathology of the blood in pernicious anæmia. Since, while pursuing investigations into this subject, he found what had not been previously noticed—viz., that when a drop of blood from a person suffering from pernicious anæmia was allowed to fall on a glass slide, and then, when the edge of the drop had dried somewhat, a cover glass was gently placed upon it, crystals of hæmoglobin gradually formed in the film of blood in from ten to forty-eight hours. This observation was of great interest, for up to that time the author had not been able to crystalise human blood, although crystals were obtained from that of lower animals. But it now seemed to the author that it would be possible to obtain crystals of human blood by imitating as much as possible the conditions which obtain in pernicious anæmia. A chief characteristic of the blood in this disease is a specific gravity some 20deg. below the normal, obviously due to the small number of red corpuscles present, the plasma therefore being in proportionally much greater quantity than in health. The effect of diluting normal blood with serum was therefore tried, serum from sheep's blood being used, since it is well known that the serum of the blood of one animal tends to destroy the corpuscles of another. At first, success did not follow, but having used serum which had been set aside for some days and had become somewhat decomposed, crystals appeared in every instance in which it was used. Blood from twenty-five members of the pathology class of St. Thomas was experimented with, and crystals were obtained in every case. Such crystals are in the form of rectangular plates of a pink colour. This, then, evidently forms a diagnostic point of great importance, as there is an universal consensus of opinion that the blood of lower animals invariably crystalises as oxyhæmoglobin only, while the crystals described above consist of reduced hæmoglobin. The only exception to this rule is found in the blood of the monkey. At first sight it might appear that the blood of the monkey, acting like human blood, was a serious objection, but it is not an insurmountable one, the shape of the crystals being different, the human crystal being rectangular, the monkey crystal diamond shaped, with, however, two long sides.

* *British Medical Journal*, July 27, 1889.

